# Treatment of Pancreatic Cancer: What Can We Really Predict Today?

**DOI:** 10.3390/cancers3010675

**Published:** 2011-02-17

**Authors:** Jean-Baptiste Bachet, Raphael Marechal, Jean-Luc Van Laethem

**Affiliations:** 1 Gastrointestinal Cancer Unit, Department of Gastroenterology, Hôpital Erasme, Université Libre de Bruxelles, 808 route de Lennik, B-1070 Brussels, Belgium; E-Mails: rmarecha@ulb.ac.be (R.M.); jean-baptiste.bachet@psl.aphp.fr (J.B.B.); 2 EA4340 “Epidémiologie et oncogènes des tumeurs digestives”, Université de Versailles, St-Quentin-en-Yvelines, France; 3 Department of Gastroenterology, Hôpital Pitié Salpêtrière, Assistance Publique—Hôpitaux de Paris, France

**Keywords:** pancreatic, cancer-personalized, therapy-predictive, biomarkers-gemcitabine, nucleoside, transporters-CXCR4-SMAD4-hENT1

## Abstract

Managing pancreatic cancer remains a big challenge due to its worse course and prognosis. However, therapeutic options and multimodal strategies are increasing nowadays, including new agents, new regimens and chemoradiation. Recently, the FOLFIRTNOX regimen has been reported to be more active than gemcitabine in selected metastatic patients. In this setting, it will be of utmost interest to guide our therapeutic choice not only on clinical and pathological findings, but also on specific biomarkers that will predict tumor behavior and patient outcome (prognostic markers), and benefit from specific agents or regimens (predictive markers). In the near future, we will have to build both our therapeutic interventions and our clinical research based on an accurate patients' clinical selection and on biomolecular markers. In this review, we aimed to highlight and discuss some of the recent results reported on biomarkers in pancreatic cancer that may predict, *i.e.*, preferential metastatic diffusion after surgery, like CXCR4, or predict gemcitabine efficacy in an adjuvant setting as well as in advanced disease, like hENT1. An important effort for translational research in pancreatic cancer research is thus required to validate such markers, while some important questions concerning tissue availability and processing, methodology of analysis, and design of future prospective trials, need to be addressed.

## 1. Introduction

The incidence of pancreatic adenocarcinoma (PAC) increases regularly in most of the western countries and this cancer ranks as the fourth cause of death by cancer [1]. Considering all stages, five years survival is less than 5% [2,3]. At diagnosis, 50% to 60% of patients have advanced disease with distant metastases, and out of the 10% of patients who undergo a curative resection, many will relapse with metastases.

In patients with resectable disease, adjuvant chemotherapy allows to improve the five year survival rate, from about 10% with surgery alone, up to 20% with post-operative chemotherapy [4-7]. In the CONKO-01 trial (Charite Onkologie), adjuvant gemcitabine was shown to significantly improve disease free and overall survival whatever resection status, nodal status or tumor size [4]. 5-Fluorouracil based chemotherapy (5FU) was as efficient as gemcitabine monotherapy in the ESPAC-3 adjuvant trial but gemcitabine was a little bit better tolerated [7]. Thus, gemcitabine or 5FU are today the drugs of choice for adjuvant chemotherapy which is indicated in all patients with a resectable PAC. Whereas, adjuvant radiochemotherapy (RCT) is still controversial, and its benefit may be restricted to patients with R1 resection [6,8].

In locally advanced disease, induction RCT has been shown to be more toxic and less effective than front-line chemotherapy [9]. Nevertheless, in the subgroup of patients with well controlled disease after a few months of front-line chemotherapy, RCT may have a place of choice, allowing to prolong overall survival and to propose treatment break [10]. This strategy is currently evaluated in an ongoing phase III trial.

In metastatic PAC, monotherapy with gemcitabine remained the main therapeutic option during more than 10 years [11]. In Phase III studies, many different combinations of drugs and new targeted therapies have been tested with gemcitabine. Unfortunately, most of these studies were negative and failed to confer any added benefit on overall survival in comparison to gemcitabine alone. Combinations of gemcitabine with fluoropyrimidine or derivative platinum were only associated with a significant improvement in overall survival in meta-analyses [12-14]. Only one study evaluating a targeted therapy reports an improvement in overal survival with a combination of erlotinib and gemcitabine, but this effect was modest with a survival benefit of less than two weeks [15]. Recently, at the 2010 ASCO meeting, FOLFIRINOX regimen, which combines the three cytotoxics 5FU, irinotecan and oxaliplatin, has shown a significant benefit on progression free survival and overall survival in comparison with gemcitabine alone [16]. Only patients with a performance status of 0–1 have been included in this study, and, despite reported as higher, grade 3–4 toxicities were manageable and no toxic death was reported in the FOLFIRINOX arm [16].

For the last 10 years, an increasing number of translational studies in PAC have been published on potentially prognostic and/or predictive makers. Because more options are available today in PAC treatment, identification of robust markers should be a priority. It may allow to choose the best option(s) for each patient and to therefore improve overall survival. Presently, two types of markers seem to be of particular interest. On the one hand, identification of markers (*i.e.* prognostic) to predict the course of the disease after surgery, either metastatic or locoregional recurrence/spread, could better define the indication of RCT. On the other hand, identification of markers (*i.e.*, predictive) of treatment efficacy or benefit, could allow to personalize the choice of chemotherapy.

The aim of this review is to highlight some recent knowledge on prognostic and predictive markers in PAC and to discuss their potential use in the different settings.

## 2. Markers that Predict Metastatic Diffusion or Recurrence Sites after Surgery

Whereas chemotherapy is a systemic treatment, it can be considered that RCT, due to the dose and schedules of chemotherapy used in combination with radiotherapy, have only a locoregional efficacy, and are not optimal to control occult distant metastases. This point is probably the major reason for the disappointing results reported with RCT in both an adjuvant setting and locally advanced disease [6,9]. Nevertheless, some results support the use of RCT in subgroups of patients, *i.e.*, those with a high risk of locoregional recurrence after R1 resection [8], and those with a locally advanced disease well controlled by first-line systemic chemotherapy [10]. Thus, identification of markers able to differentiate subgroups of patients with a higher risk of metastatic diffusion as compared to locoregional recurrence may be of great interest.

### 2.1. Issues with Positive Resection Margins after Duodenopancreatectomy

To be able to adequately interpret the prognostic significance of these putative markers, we have to correctly address and define the resection margins status, which is clearly one of the main prognostic factors of (locoregional) recurrence and outcome in pancreatic cancer [4-7].In the adjuvant setting, a large meta-analysis of five randomized trials reported that RCT was not associated with an increase in the risk of death [HR = 1.09; 95% CI: 0.89–1.32], median survivals being 15.8 months with, and 15.2 months without, RCT in the whole population [8]. In subgroup analysis, there was a significant difference in the effect of RCT dependent on R0 or R1 resection margins (*p* = 0.04); RCT being considered as more effective in patients with R1 resection. However, the rates of R1 resection reported in these five trials were very heterogeneous, varying from 17% to 82% [8]. Such heterogeneity is also observed in the literature with a large difference between series according to the centers and the procedures used for pathological examination, reflecting the absence of standardization [17-20]. In International Union Against Cancer (UICC) classification, R1 resection is referred to tumor cells observed by microscopy at one or more edges of the resection specimen [21], while the Royal College of Pathologists' guidelines defined R1 resection as the presence of tumor from 1 mm or less from the circumferential resection margin (CRM), or surface of the resection specimen, either by direct tumor invasion or lymph node involvement [22]. For anatomic reasons, and at the difference of adjacent organ and pancreatic transection margins, the CRM requires a specific pathological procedure to be correctly assessed. The Royal College of Pathologists' guidelines recommend evaluating the CRM in three different areas according to a plane perpendicular to the duodenal axis: the superior mesenteric vein groove separates the anterior CRM from the posterior CRM. In using this specific protocol, the rate of R1 resection is of about 80%, considerably higher in comparison with historical series [19,20]. However, the prognosis significance of R1 resection is probably not the same for all resection margins. In a recent retrospective study, involvement of the beds of the major mesenteric vessels and/or transection margins (n = 44, 44%) had a worse prognostic significance while there was no significant difference in survival between patients with only R1 anterior and/or posterior CRM (n = 61, 56%) compared with those with R0 resection (n = 39, 26%) [23].

Regarding the independent prognostic significance of R1 resection in randomized adjuvant trials [7,24], and its potential impact upon RCT indication, a rigorous and standardized examination of the resected pancreatic specimen is thus necessary. Moreover, as long as the prognostic significance of the different R1 resection margins is not clearly established, the real impact of others markers predicting patients' outcome cannot be adequately assessed.

### 2.2. SMAD4 and TGF-β Pathways

Transforming growth factor-β (TGF-β) signaling pathways are involved in tumor pathogenesis and progression through different effects on cell differentiation, proliferation and invasion [25]. In PAC, a global genomic analysis has reported that perturbations of TGF-β signaling were found in 100% of the 24 analyzed tumors [26]. In the same way, it was shown that TGF-β ligands and the type II TGF-β receptor were overexpressed in cDNA microarray analysis as in quantitative proteomic analysis in PAC [27-29], and enhanced expression of TGF-β receptor II has been reported as a worse prognostic factor [30].

Functionally, the binding of TGF-β ligands induces the formation of heterotetrameric active receptor complexes that regulate the activation of downstream SMAD and non-SMAD pathways. In the SMAD pathway, phosphorylation of SMAD proteins by activated TGF-β receptor complexes lead to the formation of heteromeric complexes with the common partner SMAD4, also termed DPC4. Then, activated SMAD complexes translocate into the nucleus where they interact with other transcription factors to regulate expression of various genes [31]. *SMAD4* was originally isolated from human chromosome 18q21.1 as a tumor suppressor gene, with about 55% of pancreatic cancers bearing deletions or mutations [32]. Subsequent to the demonstration that immunohistochemical labeling for SMAD4 is a sensitive and specific marker for *SMAD4* alterations, the prognostic value of SMAD4 expression has been assessed in patients with resected or locally advanced PAC [33]. However, results of the two largest published studies were discordant, SMAD4 protein expression being reported as a good prognostic factor in one [34], but, contrarily, as a worse prognostic factor in the other [35]. More recently, in another study of 89 patients with resected PAC, *SMAD4* gene inactivation (identified using high-density oligonucleotide array) was associated with shorter overall survival in multivariate analysis [36]. In fact, the population of patients included in these three studies was not comparable, particularly with regards to adjuvant treatment. *SMAD4* inactivation was reported as a worse prognostic factor in a monocentric study in which patients received adjuvant RCT [n = 249; HR = 1.36, 95% CI: 1.03–1.81, *p* = 0.042] [35], and in a second study in which most of the patients received adjuvant RCT and 5FU chemotherapy [HR = 1.92, 95% CI: 1.20–3.05, *p* = 0.006] [36]. Whereas, *SMAD4* inactivation was a good prognostic factor in a study in which only 11% of patients received 5FU adjuvant chemotherapy [34]. Consequently, interactions between *SMAD4* status and the type of adjuvant treatment may be a confounding bias and may explain the discordant results reported.

These conflicting results are not easy to understand, and may also reflect the complex network of TGF-β signaling in cancer [25,37,38]. On the one hand, TGF-β, via SMAD pathway, is a potent inhibitor of epithelial cell growth and survival through modulation of cell cycle regulators and activation of apoptosis, but these effects are highly dependent on cellular context and tumor microenvironments [37]. On the other hand, and in using others pathways, TGF-β can promote proliferation, migration, and the epithelial-to-mesenchymal transition (EMT) which is implicated in metastatic phenotype [38].

Results of a study in murine model of PAC (with *KRAS* mutation) and in cell lines, have well described the different effects of TGF-β signaling according to the presence or not of SMAD4 [39]. Briefly, *SMAD4* deletion enhanced PAC pathogenesis directed by *KRAS* mutation, but also altered the tumor phenotype: tumors with SMAD4 deficiency were well-to-moderately differentiated and expressed epithelial markers, whereas tumors with intact SMAD4 frequently exhibited markers of EMT. Moreover, cell line responses to TGF-β were different according to SMAD4 status. In cell lines from mice with SMAD4 deficiency, TGF-β had no effect on proliferation and increased modestly cell migration whereas, in cell lines from mice with intact SMAD4, TGF-β had the opposite effect according to cell differentiation: in well or moderately differentiated cells, it inhibited cell migration, cell growth, and promoted apoptosis, but it enhanced migration and increased proliferation in undifferentiated cells [39]. These results are consistent with the dual function of TGF-β pathways in cancer, and with results recently reported in breast cancer cell lines in which the type of motility (“single” *versus* “cohesive”) was different according to SMAD4 status [40].

In summary, these data support the fact that SMAD4 status may be involved in the risk of metastatic diffusion. This has been recently addressed in 76 patients who died from PAC in whom pathological features of tumors were analyzed after autopsy and correlated to the pattern of failure. In this study, the loss of SMAD4 was significantly associated with metastatic disease in comparison with local recurrence [41]. Considering the small number of patients included in this study, any definite conclusion is unlikely at this time. The exact prognostic *versus* predictive role of SMAD4/TGF beta, requires larger prospective studies.

### 2.3. S100A2

S100A2 overexpression has recently been reported as a good prognostic marker in the largest published translational study in patients with PAC (n = 601) [42]. In this study, in which 17 candidate markers have been evaluated by immunohistochemistry or *in situ* hybridization, S100A2 overexpression was the only marker significantly associated with longer overall survival [in subgroup of resected PAC, HR = 1.87; 95% CI: 1.25–2.81; *p* = 0.0024]. However, despite the number of patients included, and the use of a training set then a validation set, results reported cannot really be conclusive because patients who received or did not receive adjuvant treatments (chemotherapy and/or radiotherapy) were mixed in the analysis [42]. Thus, S100A2 seems to be a promising marker, but its real prognostic and/or predictive value should be assessed in other studies with adequate methodology. Moreover, S100A2 function in PAC pathogenesis is unknown. S100 family seems implicated in complex network and various functions. S100 proteins have been reported to be implicated in the pathogenesis of many cancers, but were suggested as tumor suppressors in some and as tumor oncogenes in others [43]. Consequently, additional studies should be conducted to try to understand the function and role of the S100A2 protein in the pathogeneis of PAC.

### 2.4. CX Chemokine R 4 (CXCR4)

Chemokines are a large cytokine subfamily of chemotactic cytokines, specifically acting on the superfamily of G-protein-coupled seven-span transmembrane receptors. Many chemokines and chemokines receptors have been described with involvement in cell differentiation, trafficking, and activation [44]. Some of them are implicated in the development or progression of cancer, in particular the chemokine CXCL12 (also termed stromal cell-derived factor-1, SDF1) which binds to two receptors, the CXC receptor 4 (CXCR4) and the CXC receptor 7 (CCR7) [45]. CXCL12 is a homeostatic chemokine, constitutively expressed in several organs, and, in particular, the liver, lung and in bone marrow. The CXCL12/CXCR4 axis seems involved in angiogenesis, metastasis, and survival; cancer cells expressing CXCR4 migrate and spread along the CXCL12 gradients [45-48].

In PAC, *in vitro* and *in vivo* data have reported that CXCR4 overexpression was frequent while normal pancreatic tissue does not express CXCR4 [47,49]. Moreover, CXCR4 was more frequently expressed in cell lines derived from distant lesions (metastases or ascites) than in those from primary tumors [47]. CXCL12 was also found in PAC tissue samples [49]. The effects of CXCL12 on CXCR4 positive cell lines have been well documented, and result in various effects: it stimulates cell migration in a chemotaxis assay, enhances cell transendothelial migration, and induces matrix metalloproteases activation [47,48]. In a murine model using nude mice and injection of cancer cells into tail vein, CXCR4 expression was associated with a dramatic increase in liver and lung metastases [48]. Most of these effects were abolished or significantly reduced with the use of an anti-CXCR4 monoclonal antibody, even in cell line which produce spontaneously CXCL12 [47,48]. Results of CXCL12 on proliferation and survival of cells expressing CXCR4 are discussed with discordant data published [47,48]. More recently, the CXCL12/CXCR4 axis was also described to be involved in pancreatic intraepithelial neoplasias with an expression frequency which increases during their progression [50]. Overall, these results suggest that an autocrine/paracrine loop involving this axis may play a major role in PAC pathogenesis and in metastatic diffusion.

Clinically, the prognostic value of CXCR4 expression has been evaluated in 71 patients with resected PAC. In multivariate analysis, CXCR4 expression was an independent and strong worse prognostic marker for overall survival [HR = 5.55; 95%CI: 1.92-12.31; *p* < 0.001] as well as lymph node metastases and undifferentiated histology. CXCR4 expression was also significantly correlated with the proliferative index (Ki67) and the type of relapse: liver metastases occurred in 59% of patients with high CXCR4 expression in their tumor, and in 28% of those with low CXCR4 (*p* = 0.016) [51].

Interestingly, recent data have reported that CXCR4 expression may be influenced by the TGF-β pathway and the phenotype of cancer cells. In a hepatocarcinoma cell line in which the presence of TGF-β induced EMT without suppressor effects, CXCR4 expression was stimulated by TGF-β [52]. In PAC stem cells (defined as CD133 positive), CXCR4 was markedly overexpressed in comparison with others cancer cells. Moreover, coculture of cancer stem cells with pancreatic stromal cells which express CXCL12 was associated with an increased migration and invasion ability of cancer stem cells, and these effects were significantly reduced by CXCR4 downregulation using RNA interference [53]. These data highlight the major role of stroma in PAC, and the necessity to try to understand the complex interactions between cancer cells and stromal cells.

In summary, CXCR4 may represent a specific marker of distant relapse risk, and appears as an attractive target for therapeutic options.

## 3. Markers that predict Adjuvant Chemotherapy Benefit

The five year overall survival of patients who had PAC resection is doubled when they receive adjuvant chemotherapy [4-7]. Adjuvant chemotherapy is efficient in all patients whatever resection margins, stages N or T, and is consequently indicated in all cases nowadays. However, five year overall survival remains relatively poor, about 20%. Recently, results of the ESPAC-3 trial have definitively demonstrated that gemcitabine and 5FU adjuvant chemotherapy have the same efficacy [7]. Thus, identification of predictive markers of efficacy or benefit of gemcitabine and/or 5FU is of major interest to individually guide the choice of adjuvant chemotherapy according to tumor phenotype or genotype. The aim of such strategy of treatment personalization could potentially contribute to increase overall survival.

### 3.1. Predictive Markers of Gemcitabine Benefit

Permeation of gemcitabine through the plasma membrane by diffusion is low and requires specialized integral membrane nucleoside transporters proteins. The Human Equilibrative Nucleoside Transporters (hENT) and the Human Concentrative Nucleoside Transporters (hCNTs) play a key role in the intracellular uptake of gemcitabine and thus are key determinants of gemcitabine efficacy. hENTs mediate equilibrative bi-directional transport nucleosides across membranes [54,55], whereas hCNTs transport nucleosides against their concentration gradients driven by sodium and/or proton coupled electro-chemical agents [56]. Among these transporters, hENT1 and to a lesser degree, hCNT3 mediates the majority of gemcitabine transport in preclinical models [57-60].

Preclinical studies, including studies involving PAC cells lines, have suggested a positive correlation between hENT1 gene expression and chemosensitivity. Cultured human cells that are either pharmacologically or genetically deficient for hENT1 exhibit resistance to gemcitabine [59], suggesting that hENT1 abundance may be used as a predictive marker for response to gemcitabine. Several small retrospective studies have evaluated whether the expression of hENT1 into the tumor correlated with patients' outcome [61,62]. Although all these retrospective studies suggested a prognostic role for hENT1 in PAC treated with gemcitabine, none of them studied controls who did not receive gemcitabine. Consequently, the true predictive value of hENT1 in gemcitabine-treated PAC (*i.e.*, the ability to identify those patients most likely to benefit from gemcitabine) could not be assessed in these studies. Recently, however, results of the translational study performed in a cohort of PAC patients from the large prospective randomized RTOG 9704 trial, support the concept of hENT1 as a predictive factor of gemcitabine benefit [63].

Beside the nucleoside transporters, several enzymes involved in gemcitabine metabolism, such as deoxycytidine kinase (dCK), cytidine deaminase, RRM1, and RRM2, may have also a key role in altering intracellular disposition of the drug and determining response to gemcitabine. These proteins have had limited evaluation as biomarkers in PAC. Single, small, retrospective, clinical studies have suggested the poor prognostic value of high levels of RRM1 or RRM2 [64,65] gene expression and low deoxycytidine kinase [66,67] protein expression in patients with pancreatic cancer treated with gemcitabine.

In our efforts to guide treatment decision for patients with PAC, we might better define gemcitabine sensitivity and resistance by an integrated analysis of the expression of dCK, hENT1 and hCNT3 in larger cohorts of patients, ideally within the context of a randomized controlled trial. In this manner, it may be possible to more precisely define those subgroups of patients who would derive particular benefit from gemcitabine, and those patients who warrant investigation with experimental therapies or should be treated with non gemcitabine-based combinations.

### 3.2. Predictive Markers of Fluoropyrimidines Benefit

#### 3.2.1. Histone Modifications

Epigenetic alterations are involved in pathogenesis and progression of many cancers with hypermethylation at specific promoters and massive DNA hypomethylation of wide genome sequences [68]. These modifications of DNA methylation profile are linked with an aberrant pattern of histone modification, and influence chromatin accessibility. The acetylation and methylation status of specific lysine residues of histones tails is known to play a major role in gene transcription, histone hypoacetylation and hypermethylaton being characteristic of methylated and repressed DNA sequences [68,69].

Such alterations have been described in PAC, resulting in expression or repression of some specific genes [70,71]. Moreover, low cellular levels of histone H3 lysine 27 trimethylation has been reported to be a worse prognostic marker [72]. Recently, the prognostic and predictive value of three histone modifications have been assessed in two cohorts of patients with PAC: 195 patients from RTOG 9704 trial, and 140 patients from Los Angeles Medical Center [73,74]. In multivariate analysis for overall survival, low H3K9me2 and H3K4me2 were independent worse prognostic markers in both cohorts. The predictive value of these markers has been evaluated in patients stratified according to histone groups. No significant difference was shown in patients subgroups with high histone levels, and this regardless of the adjuvant chemotherapy used. In contrast, in low H3K4me2 or H3K18ac subgroups, patients who received 5FU had worse disease free survival than those who received gemcitabine. Moreover, patients with low H3K4me2 or H3K18ac had a significantly worse overall survival than patients with high levels in the 5FU arm, but no difference was observed in the gemcitabine arm [73].

These results suggest that low levels of H3K4me2 or H3K18ac could be predictive markers of the absence of 5FU adjuvant benefit. This hypothesis is supported by *in vitro* data that have shown that histone deacetylase inhibitors act in synergy with 5FU to increase cytotoxicity and growth inhibition of cancer cell lines; this synergy being at least partly consecutive to downregulation of thymidylate synthase [75,76].

#### 3.2.2. Thymidylate Synthase

To our knowledge, no study has evaluated the predictive value of thymidylate synthase (TS) expression in PAC. However, results reported in colorectal cancer could lead to future research. Number mechanisms are implicated in the antitumor effect of 5FU. Among them, competitive inhibition of TS is one of the mains, and the predictive value of TS expression on 5FU sensitivity has been well described *in vitro* [77,78]. *In vivo*, a large meta-analysis has reported that a high level of TS was a worse predictive marker of overall survival in metastatic colorectal cancer [HR = 1.74; 95% CI: 1.34–2.26] as in adjuvant setting [HR = 1.35; 95% CI: 1.07–1.80] [79]. TS expression was assessed with immunohistochemistry, reverse transcriptase and/or enzyme assay in the different studies. Despite significant results, the authors recommended additional studies because of heterogeneity evidence and potential publication bias [79]. Thus, TS expression could be assessed in pancreatic tumors using various techniques.

Another possibility to assess TS predictive value is to analyze its polymorphisms. Two main genetic determinants of TS expression have been reported: the number of tandem repeat polymorphism in the *TS* promoter-enhanced region (5′UTR) [80,81], and a short insertion and deletion of 6 base pair (bp) in the 3′-untranslated region (3′UTR) [82]. Moreover, a single nucleotide polymorphism (G/C) located within the second repeat of a three repeat sequences polymorphism (in the 5′UTR) is also correlated with transcriptional activity [83]. These three markers combined were predictive of outcome in a retrospective analysis of patients with stage II or III colon cancer treated with adjuvant 5FU [84]. In addition, a recent analysis in a prospective randomized trial has reported that the 5′UTR repeat polymorphism was an independent predictive marker of response in patients treated with 5FU for metastatic colorectal cancer [85]. All these results suggest that the predictive value of TS on 5FU adjuvant benefit should be assessed in PAC.

#### 3.2.3. Microsatellite Genetic Instability Status

In colorectal cancer, at least two main tumorogenic pathways have been identified: the microsatellite instability (MSI) pathway, and the chromosomal instability (CIN) or microsatellite stability (MSS) pathway. MSI pathway occurs in approximately 15% of colorectal cancers, and its most common cause is a loss of the DNA mismatch repair function which can result, on the one hand, from germline mutation in an inherited context of Lynch syndrome (also termed: hereditary non polyposis colorectal cancer, HNPCC) or, on the other hand, from epigenetic alterations with hypermethylation of *hMLH1* promoter [86,87]. For MSI colon cancer diagnosis, independently of HNPCC screening, polymerase chain reaction (PCR) assay followed by testing of microsatellite length and immunohistochemistry tests reported approximately the same results in recent studies [88]. These results support the fact that both methods can be used in colon cancer, according to local facilities and expertise. At present, MSI is a validated robust prognostic factor in colon cancer, and there is accumulating evidence that MSI phenotype is predictive of the absence of 5FU adjuvant chemotherapy benefit [88]. Interestingly, pancreatic cancer is one of the HNPCC-related tumors in the revised Bethesda guidelines, and it is part of the criteria for testing colorectal tumors for MSI [89]. Indeed, the rate of MSI in PAC seems to be of about 15%, similar to colon cancer [90-92]. In the largest study published, some of MSI PAC resulted from germline mutation in HNPCC syndrome (3%), and most of the others resulted from *hMLH1* promoter hypermethylation [90]. In all these studies, biological diagnosis of MSI was done using PCR assay [90-92], and, to our knowledge, immunohistochemistry has never been assessed to identify MSI in PAC. Clinically, MSI phenotype has been reported as a good prognostic marker in comparison with MSS tumors in two series of PAC [90,91]. In the first, the seven patients with a MSI tumor had a significantly longer overall survival than the other 33 patients in univariate analysis (*p* = 0.0057) [90]. In the second, in which 46 patients were included (MSI tumors = 8), MSI phenotype was an independent prognostic marker in multivariate analysis [HR = 5.58; 95% CI: 1.60–19.43] [91]. In this last study, and as in colon cancer, MSI phenotype was associated with a larger intensity of tumor-infiltrating leukocyte [91,88]. The small number of patients included in the studies, and their retrospective type do not allow definitive conclusions. Nevertheless, the high similarities that seem to exist between the data in colon and pancreatic cancer could justify an evaluation of the predictive value of MSI phenotype for 5FU adjuvant chemotherapy benefit in PAC.

## 4. Predictive Markers of Palliative Chemotherapy Benefit

### 4.1. Why Will It Be of Interest?

Therapeutic options in metastatic PAC have recently increased with the results of the PRODIGE 4/Accord 11 trial that demonstrated the superiority of FOLFIRINOX regimen in comparison with gemcitabine alone [16]. Only patients with a performance status of 0–1 were included in this study, and treatment decision making could be clinically based on performance status; gemcitabine alone remaining the standard in patients with grade 2 performance status. However, apart from concerns of toxicities, FOLFIRINOX is probably efficient, whatever the performance status, and specific predictive biomarkers of this combination should therefore be evaluated. Moreover, erlotinib and gemcitabine combination was associated with a modest efficacy on overall survival in the whole study population while a subgroup of patients probably benefit from erlotinib addition [15]. The same reasoning can be applied to derivative platinum combinations [12,13]. In this context of increasing options, identification of predictive markers may be of great help to personalize the use of these drugs and to decide on the use or not of a gemcitabine-based regimen.

### 4.2. Erlotinib

Erlotinib is a human epidermal growth factor receptor (EGFR) tyrosine kinase inhibitor. In the Phase III randomised trial in patients with advanced PAC, patients who received the erlotinib and gemcitabine combination had a significantly longer overall survival than those treated with gemcitabine alone (6.24 months *vs.* 5.91 months, *p* = 0.038) [15]. In subgroups analysis, the hazard ratio for survival was significant only in the subgroup with metastatic disease [HR = 0.79; 95% CI: 0.65–0.97], and not in the subgroup with locally advanced disease [HR = 0.94; 95% CI: 0.63–1.39]. In this study, EGFR level evaluated on archival tissue by immunohistochemistry had no prognostic or predictive value in both arms. However, the occurrence of skin rash was a predictive factor of overall survival in multivariate analysis [HR = 0.74; 95% CI: 0.56–0.98] [15]; this was confirmed in another large Phase III study [93].

Normally, activation of EGFR leads to a cascade of phosphorylation in many pathways: the RAS/RAF/MAPK, PI3K/AKT, and STAT pathways which are implicated in cellular proliferation, adhesion, angiogenesis, migration, and survival [94]. Notably, mutations of genes of these pathways are frequently found in most cancers. In PAC, *KRAS* mutations are observed in 75–90% of cases [95–97], *EGFR* mutations in 1%-2%, and *EGFR* amplification in 40%-50% [97,98]. In colon cancer, the negative predictive value of *KRAS* mutation status for the anti-EGFR antibodies efficacy has been reported first in retrospective studies then definitively confirmed by retrospective analysis of tumors of patients included in large prospective trials [99,100]. In wild-type *KRAS* colon cancer, *EGFR* amplification has also been reported as a predictive marker [101]. In non-small cell lung cancers, *EGFR* mutations, wild-type *KRAS*, and *EGFR* amplification have been described as predictive marker of erlotinib efficacy [102,103].

In PAC, the predictive value of *KRAS* mutations and *EGFR* amplification for erlotinib efficacy has been assessed from tumor samples of 26% of patients included in the prospective randomized Phase III trial [97]. The hazard ratio of death between the two arms was 0.66 [95% CI: 0.28–1.57] for patients with wild-type *KRAS*, and 1.07 [95% CI: 0.28–1.57] for patients with mutated *KRAS* (interaction; *p* = 0.38). There was also no significant difference between the two arms according to *EGFR* amplification. One of the reasons for these disappointing results is the possibility of interactions between gemcitabine and erlotinib effect in the combination arm and the confounding role of *KRAS* status as prognostic or predictive.

Consequently, predictive markers of erlotinib efficacy should be assessed in patients with PAC treated by erlotinib alone, or in combination with predictive markers of gemcitabine efficacy, both in the setting of adjuvant and advanced disease clinical trials.

### 4.2. Predictive Markers of Platinum Derivatives Benefit and Toxicities

To our knowledge, no study has evaluated predictive markers of oxaliplatin and cispatin efficacy and/or toxicity in patients with PAC. However, results reported in others cancers could lead to future research.

Oxaliplatin and cisplatin act through formation of DNA adducts which interfere with DNA replication. DNA repair enzymes, in particular, proteins of the nucleotide excision repair (NER) pathway, are thought to repair DNA damage caused by platinum agents, and play a major role in preventing cell death. The excision repair cross-complementation group 1 (ERCC1) protein functions in a complex with others NER proteins, and is required for the excision of the damaged DNA [104]. *In vitro*, the expression level of *ERCC1* mRNA has been shown to be correlated with derivative platinum cytotoxicity [105,106]. Moreover, it has been suggested that the NER was implicated in the cytotoxic synergism observed between gemcitabine and cisplatin [107]. Clinically, many retrospective studies have reported a significant correlation between the expression level of *ERCC1* mRNA and the efficacy of derivative platinum based chemotherapy in colorectal, esophageal, and gastric cancers [108-110]. Furthermore, in non-small-cell lung cancer, the expression level of ERCC1 assessed by immunohistochemistry has been reported as a significant predictive marker for overall survival in a retrospective analysis of an adjuvant Phase III trial in which patients were randomized between an arm of cisplatin-based adjuvant chemotherapy and an observation arm [111].

Several polymorphisms of NER enzymes have also been described. In patients with metastatic colorectal cancer, *ERCC1* Asn118Asn (354C > T) polymorphism has been reported as a predictive marker of objective response in one study [112], and of overall survival in another [113]. However, results of two large retrospective studies in patients with colorectal cancer included in prospective trials did not confirm a predictive value of *ERCC1* codon 118 polymorphism [85,114].

Glutahione S-transferases (GSTs) are a family of Phase II detoxification enzymes implicated in cellular resistance to drugs. Several enzymes are found in this class, with various activities and tissue specificities. Out of them, polymorphisms of *GSTP1*, *GSTT1* and *GSTM1* are associated with variations in their protein activity [115]. The predictive value of these polymorphisms has recently been assessed in three retrospective analyses in patients with metastatic gastric (one study) or colorectal cancer (two studies) [85,114,116]. In two of these studies, *GSTP1* polymorphism was significantly associated with oxaliplatin-related neurotoxicity [114,116]. Whereas, the value of *GSTT1* and *GSTM1* polymorphisms was less clear [85,114,116]. Because of discordant results, additional studies in a larger cohort of patients are necessary to definitively conclude about the predictive value of these polymorphisms. In patients with metastatic PAC, their value to predict the toxicities from the FOLFIRINOX regimen should be evaluated.

### 4.3. Predictive Markers of Irinotecan-Related Neutropenia

Irinotecan is a topoisomerase I inhibitor of which the active metabolite SN38 is eliminated predominantly by glucuronidation. This reaction is principally mediated by UDP-glucuronosyltransferase 1 family polypeptide A1, which is encoded by the *UGT1A1* gene. Activity and expression level of UGT1A1 depends on polymorphisms of TA repeats number in the TATA element of *UGT1A1* gene [117]. The wild-type allele (UGT1A1*1) has six TA repeats, and the variant allele seven (UGT1A1*28). Patients who are homozygous for UGT1A1*28 have less capacity to eliminate SN38, and many studies have reported a significant increase risk of grade 3-4 neutropenia and/or diarrhea in those patients treated by irinotecan for metastatic colorectal cancer. However, a meta-analysis demonstrated that the risk of grade 3–4 neutropenia in patients with a UGT1A1*28/*28 genotype was correlated with the dose of irinotecan [118].

Irinotecan is active in pancreatic cancer cells [119] and was shown to have an activity in first- and second-line in patients with advanced PAC [120-122] Moreover, recent results of the PRODIGE 4/Accord 11 trial support the use of irinotecan in PAC [16]. To our knowledge, no study has evaluated the risk of neutropenia according to *UGT1A1* polymorphisms in patients receiving FOLFIRINOX. In this regimen, the irinotecan dose is 180 mg/m^2^. Whatever the type of regimen, the predictive value of *UGT1A1* polymorphisms on outcome has not been yet established in PAC. Considering the high number of neutropenic events observed with FOLFIRINOX in the above study, this marker can be helpful.

## 5. Predictive Markers: Ready For Use?

In this review, we aimed to highlight some of the recent results reported on predictive markers in PAC. These markers may predict preferential metastatic spread after surgery like CXCR4 [51], or predict drug efficacy/benefit in an adjuvant setting, as well as in advanced disease like hENT1 [63], and could help to guide our therapeutic options. Nevertheless, despite promising results for some of them, no predictive marker can be considered as validated today. In PAC, most of the published data have not been confirmed, and except for studies on hENT1 and histone modifications in patients prospectively included in the RTOG 9704 trial, all of them were retrospective. Furthermore, most of the predictive markers for 5FU, oxaliplatin and irinotecan efficacy/toxicity have been evaluated in other digestive cancers and not yet in PAC. An important effort for translational research in PAC is thus required while some important questions concerning tissue availability and processing, methodology of analyses, and design of future prospective trials need to be discussed and solved.

### 5.1. Issues with Tissue Sampling

At diagnosis, only 10% to 15% of patients with a PAC can have a curative resection, and these resected specimens constitute the best samples for translational research, both quantitatively and qualitatively, allowing analyses at the DNA, RNA, or protein level. Consequently, with the exception of patients with locally advanced tumor who had an exploratory laparotomy with tumor biopsies, most recent studies which reported prognostic and/or predictive markers have only included patients with resected PAC. However, considering that the large majority of patients have an advanced disease at diagnosis, new techniques to provide sufficient quantity/quality tumors samples should be developed in those patients. Such progress could allow inclusion of more patients in translational research studies, to accelerate them, and to assess if results reported in patients with resected tumors are valuable in patients with advanced tumors. In this way, development of robust and reproducible methods to obtain tumor samples from endoscopic ultrasound-guided fine-needle aspirations or biopsies, or from transparietal biopsies should be a priority [123]. Furthermore, besides cytopathologic samples, evaluation of systemic blood markers such as miRNA or SNPs, seem promising [124,125].

### 5.2. Issues concerning Methodological Analyses and Interpretation

As we discussed earlier, most recent studies which reported prognostic markers have included patients with resected PAC. However, because of the relatively few number of resectable patients, most of these studies have included a mixed population of patients who received various adjuvant treatments and who were resected or not. Indeed, a prognostic marker discriminates a population of patients by the overall survival in the absence of treatment, or despite it, and consequently, the best setting to evaluate a prognostic marker remains a population of patients who did not receive adjuvant treatment. However, a predictive marker separates a group of tumors or patients by the response or resistance to a specific treatment. The best method to assess the predictive value of a marker for a specific treatment is to evaluate the benefit of this treatment in the different subgroups of patients stratified for this marker. For the assessment of the prognostic value of a marker, analyzing a mixed patients' population who received or did not receive adjuvant treatment can therefore result in discordant results and interpretation. As an example, microsatellite instability (MSI) phenotype, which is a good prognostic marker but a negative predictive marker of adjuvant 5FU benefit in stage II-III colon cancer [126,127], has initially and, surprisingly, been reported as a positive predictive marker of adjuvant 5FU benefit [128]. In this first study, authors compared patients with MSI phenotype tumors to those with microsatellite stability (MSS) tumors in subgroups defined by adjuvant 5FU administration. In the subgroup of patients who did not receive adjuvant 5FU, no survival difference was observed between MSI and MSS tumors (5-year survival: 37% *vs.* 32%; *p* = 0.82) whereas in the subgroup of patients who received adjuvant 5FU, patients with MSI tumors had a significantly better five year survival than those with MSS (90% *vs.* 35%; *p* = 0.0007) [128]. In this article, there is no clear explanation for the absence of MSI prognostic value in patients who did not receive adjuvant 5FU but this result was confounding. Whereas, the better survival of patients with MSI tumors in subgroup treated with adjuvant 5FU was certainly due to the good prognostic value of MSI and not to a predictive one. Subsequently, most studies that further evaluated the benefit of adjuvant 5FU in subgroups of patients, defined by MSI/MSS phenotype, showed that MSI phenotype was a negative predictive marker of adjuvant 5FU [126,127].

Prospective randomized clinical trials remain the gold standard to validate prognostic and/or predictive markers and avoid confounding interpretations on their relative signification depending on the setting of treatment [129].

### 5.3. New Therapeutic Trials

During the last decade, Phase III randomized trials which evaluated cytotoxic combinations or targeted therapies in advanced PAC have accumulated negative results. Consequently, it seems today essential to modify our practice and to perform well-designed Phase II trials to identify new drugs or combinations that will harbor a high chance of success in subsequent Phase III studies [130]. Ideally, such studies should take into account our knowledge of PAC carcinogenesis, involved signaling pathways and potential predictive markers. For this, neoadjuvant Phase II trials in resectable patients are promising and may rapidly and safely discover signals of efficacy of an experimental regimen. During the window interval between diagnosis and surgery, the administration of a short neoadjuvant chemotherapy course would allow to evaluate treatment activity and constitute a test for subsequent adjuvant chemotherapy. Moreover, analysis of consecutive modifications in tumor and stroma could contribute to the identification of new targets or biomarkers, and to progress in our understanding of this disease. In the same way, prospective evaluation of predictive and/or prognostic markers should be an integral part of clinical trials in PAC with potentially a collection of tumors or blood samples from all enrolled patients [130].

## 6. Conclusions

In the near future, we will have to build both our therapeutic interventions and our clinical research in pancreatic cancer based on an accurate patients' clinical selection and on prognostic/predictive biomolecular markers. Some of the recent results reported promising data on biomolecular markers that may predict preferential metastatic spread after surgery like CXCR4 or gemcitabine efficacy in an adjuvant setting as well as in advanced diseases like hENT1. These data deserve prospective evaluation that should be an integral part of clinical trials in PAC with a standardized collection of tumors and blood samples. Most likely, the emerging new targeted therapies and new strategies will be more adequately evaluated when considered in the setting of a personalized molecular driven approach.
